# Combined Depth and Subdural Electrodes for Lateralization of the Ictal Onset Zone in Mesial Temporal Lobe Epilepsy with Hippocampal Sclerosis

**DOI:** 10.3390/brainsci13111547

**Published:** 2023-11-03

**Authors:** Junhyung Kim, Joong Koo Kang, Sang Ahm Lee, Seok Ho Hong

**Affiliations:** 1 Department of Neurological Surgery, Asan Medical Center, University of Ulsan College of Medicine, Seoul 05505, Republic of Korea; jhkim2ns@gmail.com; 2 Department of Neurology, Asan Medical Center, University of Ulsan College of Medicine, Seoul 05505, Republic of Koreasalee@amc.seoul.kr (S.A.L.); 3 Ace Neurology Clinic, Seoul 05616, Republic of Korea

**Keywords:** mesial temporal lobe epilepsy (MTLE), hippocampal sclerosis (HS), intracranial electroencephalography (EEG), anterior temporal lobectomy (ATL), amygdalohippocampectomy

## Abstract

(1) Objective: This study aimed to explore the efficacy of conventional invasive techniques in confirming unilateral seizure onset localization in mesial temporal lobe epilepsy with hippocampal sclerosis (MTLE-HS) and to investigate the association between electrode type and intracranial electroencephalography (EEG) pattern. (2) Methods: This retrospective study encompasses patients diagnosed with MTLE-HS who underwent an invasive study prior to an anterior temporal lobectomy (ATL). Intracranial EEG features were assessed for 99 seizure events from 25 selected patients who achieved seizure remission with ATL after an invasive study using bilateral combined depth and subdural electrodes. Their findings were compared to those of 21 seizure events in eight patients who exhibited suboptimal seizure outcomes. (3) Results: For the distribution of electrodes that recorded the ictal onset, hippocampal depth electrodes recorded 96% of all seizure events, while subdural electrodes recorded 52%. Among the seizures recorded in subdural electrodes, 49% were localized in medial electrodes, with only 8% occurring in lateral electrodes. The initiation of seizures exclusively detected in hippocampal depth electrodes was associated with successful seizure remission, whereas those solely recorded in the lateral strip electrodes were often linked to refractory seizures after ATL. (4) Conclusions: These findings emphasize the importance of employing a combination of depth and subdural electrodes in invasive studies for patients with MTLE-HS to enhance the accuracy of lateralization. This also cautions against sole reliance on subdural electrodes without depth electrodes, which could lead to inaccurate localization.

## 1. Introduction

Mesial temporal lobe epilepsy with hippocampal sclerosis (MTLE-HS) is an etiology-specific epilepsy syndrome primarily diagnosed based on the identification of structural abnormalities in neuroimaging studies [1]. While MTLE-HS represents the most common type of surgically treated epilepsy in adults, failure in seizure control or recurrence still occurs in a subset of patients [2]. The causes of surgical failure after anterior temporal lobectomy (ATL) for MTLE-HS are multifaceted and often rooted in the misidentification of the epileptogenic focus prior to surgery. These causes encompass a wide range of hypothetical situations, including the presence of bilateral foci [3], ipsilateral extratemporal foci [4,5], or dual pathologies [6], as well as erroneous lateralization of mesial temporal seizure origin. Addressing these challenges during presurgical evaluation and refining the surgical approach are essential steps towards improving the outcomes of ATL for MTLE-HS.

Efforts to reduce such failures and optimize surgical results remain a prominent clinical concern in the field of epilepsy management. There has been a compelling argument for considering a straightforward resection for MTLE-HS based on noninvasive lateralization, making conventional invasive electrode insertion less desirable [7,8,9]. However, in cases where there is uncertainty regarding the lateralization of the epileptogenic focus or suspicion of foci beyond the mesial temporal region, it is common practice to conduct invasive studies. While the specific indications for invasive studies might differ across epilepsy centers, presurgical invasive studies become necessary in approximately 30–40% of patients to precisely localize the epileptogenic zone [10].

The types of intracranial electrodes include parenchymal depth electrodes, subdural grid or strip electrodes, and a combination of both depth and subdural electrodes [11,12,13]. Furthermore, there is a growing trend towards the utilization of stereo-EEG (SEEG), which employs multiple parenchymal electrodes to delineate the three-dimensional aspects of the epileptogenic focus [14]. The choice of electrode type is determined by the expertise and experience of each epilepsy center, weighing the advantages and disadvantages of each electrode configuration [15]. Nevertheless, the optimal strategy for lateralizing the ictal onset zone in MTLE-HS remains not fully defined.

In this study, we summarized our institutional experiences with invasive studies and resective surgery for MTLE-HS over the past three decades. We particularly focused on the cases where bilateral combined depth and subdural electrodes were employed to confirm unilateral localization. This study aims to provide an overview of the intracranial EEG features of conventional invasive techniques, thereby pointing out both their ongoing roles and limitations.

## 2. Material and Methods

### 2.1. Study Population

**Figure 1:**
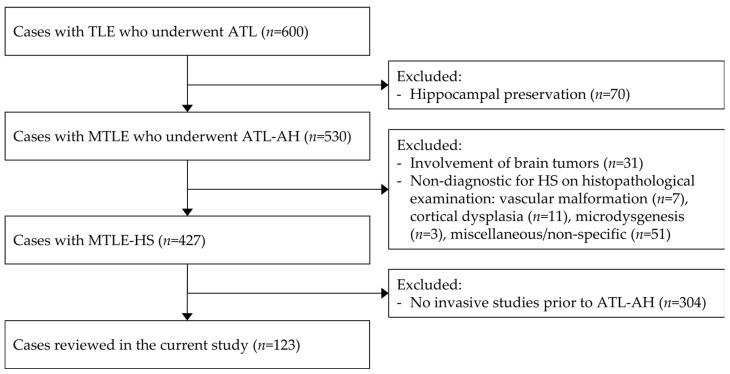
Study population. This cohort consists of individuals with histopathologically confirmed MTLE-HS who underwent invasive electrophysiological studies prior to ATL-AH at a single institution between 1994 and 2022. Invasive studies were conducted in nearly one-third (29%) of the cases. MTLE-HS, mesial temporal lobe epilepsy with hippocampal sclerosis; ATL-AH, anterior temporal lobectomy with amygdalohippocampectomy.

This retrospective study encompassed patients with MTLE-HS who underwent ATL after intracranial EEG monitoring between 1994 and 2023 at a tertiary-level referral center (Figure 1). Patients with MTLE who exhibited radiological findings of hippocampal sclerosis or atrophy with histopathological confirmation were selected for inclusion. Those with coexisting long-term epilepsy-associated tumors or with histopathological evidence indicating the absence of HS were excluded.

### 2.2. Clinical Assessment and Surgical Interventions

#### 2.2.1. Presurgical Evaluations

Patients underwent a comprehensive presurgical evaluation, which included continuous scalp and sphenoidal video-EEG monitoring, structural magnetic resonance imaging (MRI), ictal and interictal single-photon emission computerized tomography (SPECT), and [^18^F]-fluorodeoxyglucose positron emission tomography (FDG-PET). Each case was thoroughly reviewed and discussed by a multidisciplinary epilepsy team, comprising epileptology specialists, neurosurgeons, and neuroradiologists. If non-invasive evaluations did not yield concordant results regarding the lateralization or localization of the epileptogenic focus, further invasive studies were planned. Patients also underwent functional studies including functional MRI and the intracarotid sodium amobarbital procedure once the decision was made to proceed with either additional invasive study or resective surgery.

#### 2.2.2. Surgical Procedures for Invasive Studies

Intracranial electrodes were inserted using standardized neurosurgical procedures, which involved the stereotactic insertion of depth electrodes into the mesial temporal region and/or the subdural placement of strip or grid electrodes covering the temporal cortex. The 8-channel depth electrodes were sagittally inserted along the long axis of the hippocampus through an occipital entry point using frame-based stereotaxy. The decision to implant subdural electrodes was typically based on suspicion that seizures were of neocortical origin.

**Figure 2:**
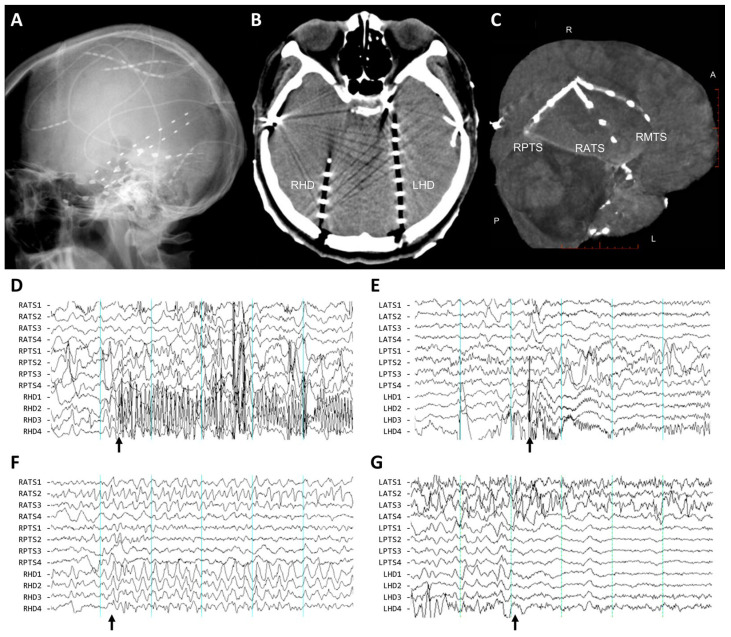
Conventional invasive electrophysiologic studies using combined bilateral depth and subdural electrodes. (**A**) A lateral skull plain radiograph depicts the placement of subdural strip and depth electrodes. (**B**) A CT scan at the midbrain level displays the bilateral insertion of the depth electrodes, reaching the hippocampus and amygdala through occipito-temporal trajectories. (**C**) A three-dimensional reconstruction of the brain CT, with contacts co-registered on the brain surface, demonstrates the standard positioning of three subdural strip electrodes covering the temporal cortex, in the anterolateral, medial, and posterolateral directions, respectively. (**D**–**G**) Electrographic patterns at ictal onset in intracranial electrodes are shown: fast spike trains (**D**), low-voltage fast activity (**E**), rhythmic slow activity (**F**), and electrodecremental patterns with diffusely flattened background activity (**G**). Each division represents 1 s (depicted in cyan lines), and calibration is set to 50 μV. Electrode contacts were numbered from deep to superficial and referenced to Pz scalp electrode. The arrows indicate the onset of each seizure event. RATS/LATS, right/left anterolateral temporal strip electrode; RPTS/LPTS, right/left posterolateral temporal strip electrode; and RHD/LHD, right/left hippocampal depth electrode.

For subdural electrodes, two or three 4-channel strip electrodes were placed via temporal craniostomy (Figure 2A–C). The medial subdural electrodes were aimed towards the hippocampus, positioned beneath the parahippocampal or fusiform gyrus, to provide comprehensive coverage of the mesial temporal surface. The lateral subdural electrodes were positioned over the temporal convexity, spanning across the lateral temporal gyri. In cases requiring extensive epicortical coverage for potential neocortical resection, a frontotemporal craniotomy was performed on the affected side, followed by the placement of two 4 × 8-channel subdural grid electrodes. The locations of the electrode contacts were determined through anatomical mapping with postoperative computed tomography (CT) scans.

#### 2.2.3. Clinical Assessment of Intracranial EEG Recording

Patients were admitted to the epilepsy monitoring unit (EMU) for continuous video-intracranial EEG monitoring one day after surgical implantation. Patients underwent monitoring for a minimum of five days and up to three weeks in the EMU. Patients discontinued or reduced their anti-seizure medication (ASM) regimens as necessary. After recording a sufficient number of spontaneous habitual seizures, the results of clinical EEG assessments were thoroughly reviewed and deliberated upon by the interdisciplinary epilepsy team, which then determined the most appropriate therapeutic options.

All clinical seizures were captured using a 64-channel digital monitoring system with a 200 Hz sampling rate. The recordings were displayed using a high-pass filter at 0.3 Hz, a low-pass filter at 70 Hz, and a notch filter at 60 Hz. Intracranial EEG recordings during the ictal onsets were visually assessed in both bipolar and referential montages. Each event was annotated for the presence of preceding epileptiform discharges, ictal onset frequencies and patterns (Figure 2D,E), the distribution of electrodes, and interhemispheric propagation time. Preceding epileptiform discharges were identified as single or periodic spikes or sharp waves at frequencies below 2 Hz before transitioning to sustained rhythmic discharges [16]. Ictal onset was marked by a localized, rhythmic EEG pattern exceeding 2 Hz that was visually different from background activity, unrelated to arousal states, and paired with clinical manifestations of the usual habitual seizures as described by patients and caregivers.

The distribution of electrodes was determined by the identification of the location that exhibited the earliest activity within the first second of ictal onset for each seizure, which was categorized as follows: (1) depth electrode only, (2) depth and medial strip electrodes (limited to the depth electrode and the first or second contacts of the medial strip electrode), (3) lateral strip electrodes (with or without the third or fourth contacts of the medial strip electrode), and (4) diffuse widespread (involving both depth and strip electrodes).

#### 2.2.4. Surgical Treatment and Outcome Evaluation

Subsequent surgical resection was carried out based on the judgments derived from the invasive study that confirmed lateralization of ictal onset. Patients proceeded with standard ATL procedures, which typically involved the resection of the anterior parts of the temporal lobe up to 4.5 cm from the temporal tip, followed by resection of the parahippocampal gyrus for amygdalohippocampectomy. In instances where the dominant hemisphere was involved, the extent of the ATL was limited to within 3.5 cm. The resected amygdala and hippocampus were sent separately for histopathological examination.

Postoperatively, patients usually continued their ASMs and underwent routine follow-up with epileptology specialists for ASM adjustment. Surgical outcomes were assessed using the Engel classification, and seizure remission was defined as outcomes corresponding to Engel Ia or Ib.

### 2.3. Characterization of the Ictal Onset Patterns of Depth and Subdural Electrodes

To explore conventional invasive study techniques, this study specifically examined a subset of patients in which both depth and strip electrodes were bilaterally inserted, and unilateral lateralization of the ictal onset zone was confirmed to be concordant with radiological findings of hippocampal sclerosis or atrophy. In this analysis, we exclusively included patients who necessitated invasive studies due to inconclusive or discordant laterality findings from the noninvasive studies.

We selected representative cases in which surgical resection was undertaken solely based on the determination of unilateral focal onset at the mesial temporal region, as ascertained through comprehensive invasive EEG monitoring. In these cases, lateralization was confirmed as the true epileptogenic foci as the patients successfully achieved seizure remission following ATL. Those who experienced complications after electrode placement that could potentially affect seizure occurrence were excluded from the analysis.

For each case review, demographic and clinical information, such as age, sex, seizure semiology, noninvasive and invasive electrophysiologic findings, structural and physiologic imaging findings, and histopathological findings, were investigated. Seizure types were categorized according to the 2017 International League Against Epilepsy (ILAE) classification [17].

### 2.4. Statistical Consideration

Descriptive data are presented as medians with interquartile ranges (IQR) unless otherwise specified. For a subset of patients who had bilateral combined depth and subdural electrode insertion, the intracranial EEG findings of the recorded seizures and patient factors were compared between those who achieved seizure remission and those who remained refractory. The baseline characteristics of selected cases were assessed for each outcome group using the standardized mean difference (Cohen’s d), where a difference of less than 0.5 was considered negligible. Groupwise comparisons of each EEG feature were analyzed using the Fisher’s exact test, with a *p*-value of less than 0.05 considered statistically significant. To account for multiple comparisons, a Bonferroni correction was applied to the *p*-values. The prognostic value of each EEG feature was reported as an odds ratio with a 95% confidence interval (CI). All statistical analyses were conducted in Python and R environments.

## 3. Results

### 3.1. Clinical Characteristics of MTLE-HS

We identified 123 patients with MTLE-HS who underwent invasive studies prior to ATL-AH. In this cohort, the average age at the time of surgery was 33.2 (SD ± 10.1) years, with 57 (46%) of them being male. The majority of patients (80%) were diagnosed with a single pathology of HS, while the remaining had a dual pathology with either cortical dysplasia (*n* = 23) or microdysgenesis (*n* = 2). For invasive studies, bilateral depth electrodes were utilized in 83% of cases (*n* = 97), while the remaining cases involved the use of strip (*n* = 20) or grid (*n* = 2) electrodes without depth electrodes. The remaining four cases underwent multiple bilateral parenchymal electrode placements for SEEG. Among those using conventional depth electrodes, most cases (*n* = 92) were combined with subdural strip or grid electrodes.

We conducted a comprehensive review of intracranial EEG data recorded for 99 seizure events from 25 selected patients (Table 1). These patients were carefully chosen among those who achieved seizure remission with ATL after an invasive study using bilateral combined depth and subdural electrodes. All patients underwent a single session of intracranial EEG monitoring. For comparison, 21 seizures from eight patients with refractory epilepsy who exhibited suboptimal seizure outcomes (two cases with Engel II, four with Engel III, and two with Engel IV) were retrieved. The baseline characteristics between the two subject groups were comparable, while the refractory cases demonstrated an earlier seizure onset age and a more prolonged disease duration: age at surgery (year), 31 (28–38) vs. 35 (29–36) (d = 0.072); age at seizure onset (year), 15 (6–26) vs. 12 (8–16) (d = 0.517); disease duration (year), 15 (11–19) vs. 19 (15–27) (d = −0.575); male, 12 (46%) vs. 5 (63%) (d = 0.320); risk factors of febrile convulsion, encephalitis/meningitis, or trauma, 17 (65%) vs. 5 (63%) (d = 0.059); and hippocampal atrophy in structural MRI, 21 (81%) vs. 6 (75%) (d = 0.139).

### 3.2. Electrographic Patterns at Ictal Onset

Out of the 99 seizure events from the 25 patients recorded in the true epileptogenic zones, preceding epileptiform discharges were noted in 49% of the seizure events (Table 2). The waveform patterns at ictal onset included a fast spike train (38%), followed by low-voltage fast activities (33%), electrodecremental activities (16%), and rhythmic slow activities (12%). Interhemispheric propagation was observed in 71 events, with 9 of them exhibiting early propagation within the initial five seconds.

Conversely, in the 21 seizures from refractory patients who failed seizure remission through ATL, preceding epileptiform discharges and fast spike train patterns were less frequently observed, occurring in 5 (24%) and 4 (19%) seizures, respectively. This implies that the absence of preceding epileptiform discharges might be associated with less favorable outcomes for resective surgery, even though it did not reach statistical significance (*p* = 0.052). An ictal onset frequency greater than 8 Hz was significantly more prevalent in cases that achieved seizure remission (*p* = 0.017). No other notable associations were identified between seizure outcomes and electrographic ictal onset patterns.

### 3.3. Distribution of Involved Electrodes and False Lateralization

In patients who achieved seizure remission through ATL, seizures were primarily detected with depth electrodes alone in 47 events and with a combination of depth and medial strip electrodes in 44 events. In three events, seizures were detected solely with lateral strips, and in five events, seizures were distributed diffusely across depth and strip electrodes. Collectively, depth electrodes recorded 96% of seizure events, whereas subdural electrodes captured 52% of them. To delineate further, subdural medial strip electrodes recorded 49% of seizure events, while lateral strip electrodes recorded 8%.

Contrarily, the electrode distribution in refractory cases presented a distinct pattern. Only two events (10%) were solely detected with depth electrodes, while nine events (43%) were captured with a combination of depth and medial strip electrodes. Eight events (38%) were detected with lateral strip electrodes, and the remaining two events (10%) showed diffuse involvement across both depth and subdural electrodes. Overall, depth electrodes captured 13 events (62%), while subdural electrodes, either medial or lateral strips, recorded 19 events (90%).

**Figure 3:**
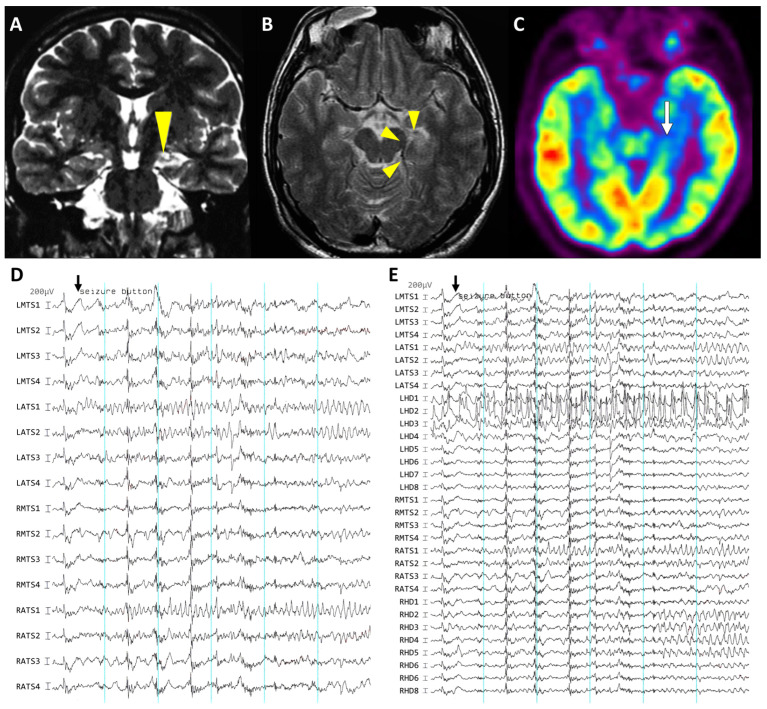
A case exhibiting discordant lateralization between hippocampal depth and subdural strip electrodes. A 28-year-old right-handed male (Case #11) first experienced seizures at the age of 17, with no prior history of febrile convulsion, head trauma, central nervous system infections, or familial epilepsy history. His seizures intensified over time to two or three significant episodes each month, even while on ASMs. These seizures initiated with an indescribable aura accompanied by an unpleasant sensation and transitioned to episodes with unresponsiveness, fumbling hand movements, repeated spitting, and drooling, which were consistent with the typical semiology of temporal lobe epilepsy. The initial MRI exhibited left hippocampal sclerosis, as indicated by arrowheads in (**A**,**B**), and a FDG-PET scan indicated hypometabolism in the left mesial temporal region, as indicated by arrow in (**C**). Noninvasive scalp video-EEG monitoring recorded 10 stereotyped seizures. However, it failed to provide clarity on lateralization, and no identifiable interictal epileptiform discharges were detected. During invasive monitoring after the insertion of bilateral combined depth and strip electrodes, the lateral strip electrodes exhibited bilateral ictal discharges (**D**). However, the hippocampal recordings clearly indicated the ictal onset on the left side, which later propagated to the right side (**E**). Following a left ATL, the patient remained seizure-free (Engel I) for over five years of the follow-up.

This result suggests that the detection of seizures via depth electrodes is predominantly associated with favorable seizure outcomes, accounting for 88% (96 of 109 events) with an odds ratio of 19.7 (95% CI, 4.6 to 83.8). Conversely, the absence of seizure detection via lateral strip electrodes was associated with seizure remission following ATL, with 89% (92 of 103 events) and an odds ratio of 10.5 (95% CI, 3.4 to 32.1). In essence, the use of subdural strip electrodes alone could lead to false lateralization in the absence of depth electrodes. Within our series, we identified three cases (Case #11, #14, and #24) of potential pseudolateralization of strip electrodes (Figure 3). In these cases, the epileptiform discharges were frequently observed unilaterally on the hippocampal depth electrodes but bilaterally on the subdural electrodes.

## 4. Discussion

In MTLE-HS, lateralization of the ictal onset zone is crucial for devising an effective therapeutic strategy. The consequences of an unsuccessful initial operation are profound, as bilateral resective surgery is not often a viable consideration due to the neuropsychological consequences it may lead to. Previous studies have suggested that suboptimal outcomes in ATL for MTLE could be partly attributed to the presence of dual pathologies in addition to HS [18]. In our cohort, histopathological examination confirmed that 20% of cases had concurrent pathologies such as cortical dysplasia and microdysgenesis. Therefore, comprehensive invasive studies should be thoroughly considered in cases where there is any discordance in lateralization or uncertainty regarding seizure semiology, imaging findings, or any other clinical suspicion of the potential involvement of other epileptogenic foci.

The insights from intracranial EEG monitoring differ from those acquired through non-invasive scalp recordings. Regarding the clinical utility of invasive study techniques, numerous reports have consistently highlighted discrepancies in localization between scalp and intracranial EEG recordings [19,20,21,22,23]. We have also encountered several cases with discordant findings between depth, subdural, and scalp recordings. The depth electrodes primarily capture hippocampal activity, while subdural electrodes cover the temporal neocortex [11]. Our results indicate that favorable surgical outcomes correspond to depth-specific ictal onsets, affirming past knowledge linking local electrographic activities and hippocampal cell loss to positive surgical outcomes [24]. Conversely, lateral strip-exclusive or diffuse widespread patterns were likely associated with unfavorable outcomes. This implies that patients with presumed MTLE-HS showing ictal onset on subdural electrodes may be susceptible to failed seizure control with ATL, despite the conspicuous presence of HS on structural MRI. Incorporating both depth and subdural electrodes in MTLE patients facilitates a better understanding of both ictal onset and propagation patterns, and such insights would remain elusive if relying solely on one type of technique [25].

The ictal onset patterns in intracranial EEG findings have been proposed as potential predictors of seizure outcomes following resective surgery [26,27,28,29]. In line with the existing literature, certain EEG features, such as a high ictal onset frequency, were linked to favorable seizure outcomes following ATL. However, some other findings from a limited number of cases in the current study seem to diverge from the prevailing literature. In our MTLE series, seizures originating from the mesial temporal region often initiated with fast spike trains and were predominantly favorable for ATL. The low-voltage fast activity, which has been considered indicative of the recording’s proximity to the epileptogenic focus and thus a potential predictor of higher benefits from resective surgery, was not evident in our findings. This suggests that these morphological ictal onset patterns may not be solid predictors of surgical outcomes. Nonetheless, further validation is needed as the current study lacked investigations on balanced controls for comparative analysis.

In terms of surgical techniques for epicortical EEG recordings, the application of three strip electrodes via a small temporal craniostomy window has the advantage of minimizing the risk of postoperative hemorrhagic complications, which frequently occur during subdural grid insertion. Meticulous surgical attention can further reduce the complication rate [30]. Some institutions have utilized an epidural grid electrode technique, which shares a similar application [31]. These techniques can be strategically combined with parenchymal depth electrodes, as they provide a clear demarcation of the resection boundary, especially in cases anticipating neocortical resection [13,32]. On the other hand, the emerging SEEG approach typically involves multiple perpendicular electrode insertions, which poses a heightened risk of intracranial vasculature violation [33]. Nevertheless, in conditions like temporal-plus epilepsy, where the operculo-insular foci cannot be ignored as a potential cause of residual seizures after ATL, a comprehensive SEEG investigation might be beneficial [29]. Therefore, the decision to utilize perpendicular depth electrodes should be carefully weighed for each candidate based on their risk-benefit profile, based on imaging findings of other structural abnormalities as well as risk factors such as febrile convulsion and status epilepticus [34].

The current study has a limitation in that it lacks exploration of the comparison between conventional techniques and SEEG in the context of efficacy, safety, and cost-effectiveness. However, we demonstrated that traditional concepts in invasive studies for epilepsy surgery maintain their efficacy, particularly in typical MTLE-HS cases. The combined utilization of hippocampal depth electrodes and temporal strip electrodes offers reliable accuracy in lateralizing MTLE epileptogenicity. Furthermore, a thorough interpretation of intracranial EEG patterns may help predict surgical outcomes. Future investigations into evaluating the clinical value of epileptic network analysis using these methodologies in comparison with contemporary SEEG approaches would be of considerable interest.

## 5. Conclusions

Conventional invasive techniques utilizing a combination of depth and subdural electrodes remain viable for the lateralization of MTLE-HS. The findings suggest caution against sole reliance on epicortical recording without depth electrodes, which could result in false lateralization, potentially affecting surgical decisions and outcomes.

## Figures and Tables

**Table 1:** Demographics and clinical characteristics of MTLE-HS cases reviewed for intracranial EEG recordings (*n* = 25).

**Case**	**Sex/Age**, year	**Onset Age (Duration)**, year	**Handedness**	**Risk** **Factors**	**Seizure** **Frequency**, /mo	**Seizure Type †**	**Preoperative Imaging Studies**			**Invasive Study**		**Surgical** **Intervention**	**Dual** **Pathology**
							**Structural** **Abnormality**	**Perfusion** **(Ictal SPECT)**	**Metabolism** **(FDG-PET)**	**Duration**, day	**Number of Events ‡**		
1	F/42	6 (36)	Lt.	Enceph.	1–2	FBTCS	Lt. HA	.	.	12	3 (3)	Lt. ATL	.
2	M/34	17 (15)	Rt.	FC	1–2	FBTCS	Rt. HS	.	.	5	3 (1)	Rt. ATL	.
3	F/38	29 (9)	Lt.	.	1–2	FBTCS	Lt. HS	.	.	7	5 (2)	Lt. ATL	.
4	M/17	5 (12)	Rt.	FC	1–2	FIAS	Rt. HA	↑Rt. T	.	3	4 (0)	Rt. ATL	CD
5	M/31	11 (20)	Rt.	.	>4	FBTCS	Rt. HS	↑Lt. T	.	5	6 (4)	Rt. ATL	.
6	M/29	14 (15)	Rt.	FC	1–2	FBTCS	Lt. HS	.	.	7	2 (2)	Lt. ATL	.
7	M/44	31 (13)	Lt.	FC	3–4	FBTCS	Lt. HS	↑Lt. T	.	6	5 (4)	Lt. ATL	.
8	F/28	13 (15)	Rt.	Enceph.	3–4	FIAS	Lt. HS	↑Lt. T	↓Lt. T	6	5 (0)	Lt. ATL	.
9	M/21	2 (16)	Ambidex.	FC	<1	FBTCS	Rt. HS	.	↓Lt. T	7	2 (1)	Lt. ATL	.
10	M/28	18 (10)	Rt.	.	2–3	FBTCS	Lt. HS	↑Lt. T	↓Lt. T	4	5 (5)	Lt. ATL	.
11	F/35	34 (1)	Rt.	.	3–4	FBTCS	Lt. HS	↑Rt. T	↓Rt. T	5	6 (4)	Lt. ATL	CD
12	M/21	9 (12)	Lt.	FC	2–3	FBTCS	Lt. HS	.	.	6	3 (2)	Lt. ATL	CD
13	F/30	3 (27)	Rt.	Trauma	1–2	FBTCS	Rt. HS	↑Lt. T	.	5	5 (5)	Rt. ATL	.
14	F/54	35 (19)	Rt.	Enceph.	1–2	FBTCS	Lt. HS	.	.	4	3 (1)	Lt. ATL	.
15	F/22	5 (25)	Rt.	.	1–2	FBTCS	Rt. HS	.	↓Rt. T	6	3 (3)	Rt. ATL	CD
16	M/39	16 (24)	Rt.	Enceph.	1–2	FIAS	.	.	.	6	5 (0)	Rt. ATL	.
17	F/18	6 (12)	Rt.	FC	>4	FBTCS	Lt. HS	↑Rt. T	.	7	3 (3)	Lt. ATL	.
18	M/28	25 (3)	Rt.	FC	>4	FBTCS	Rt. HS	↑Rt. T	↓Rt. T	3	5 (5)	Rt. ATL	.
19	M/34	24 (11)	Rt.	FC	>4	FBTCS	Rt. HS	↑Rt. T	↓Rt. T	6	3 (2)	Rt. ATL	.
20	M/29	14 (15)	Rt.	.	1–2	FBTCS	.	↑Rt. T	↓Rt. T	7	3 (3)	Rt. ATL	.
21	F/36	26 (10)	Ambidex.	.	>4	FBTCS	.	↑Rt. T	↓Rt. T	3	5 (5)	Rt. ATL	MD
22	F/53	39 (14)	Rt.	FC	2–3	FBTCS	.	.	↓Rt. T	6	5 (5)	Rt. ATL	.
23	F/37	5 (32)	Rt.	FC	1–2	FBTCS	Rt. HS	↑Rt. T	↓Rt. T	6	3 (3)	Rt. ATL	.
24	F/27	1 (19)	Lt.	FC	2–3	FBTCS	Rt. HA	.	.	6	4 (4)	Rt. ATL	.
25	F/31	26 (4)	Ambidex.	.	3–4	FBTCS	Lt. HS	.	.	7	3 (3)	Lt. ATL	.

† Most common seizure characteristics. ‡ Total number of recorded seizures (number of secondary generalization). ‘↑’ and ‘↓’ represents an increase or decrease in the perfusion or metabolism, respectively. Ambidex., ambidextrous; Enceph., encephalitis; FC, febrile convulsion; FIAS, focal impaired awareness seizure; FBTCS, focal to bilateral tonic–clonic seizure; HA, hippocampal atrophy; HS, hippocampal sclerosis (hippocampal atrophy with T2 hyperintense signal change); T, temporal cortex; ATL, anterior temporal lobectomy; CD, cortical dysplasia; and MD, microdysgenesis.

**Table 2:** Electrographic features observed during invasive studies using bilateral combined depth and subdural electrodes.

	Seizure Events		Cases †	
	Seizure Remission	Refractory	Seizure Remission	Refractory
***N***	99	21	25	8
**Preceding epileptiform discharges**				
Present	49 (49)	5 (24)	17 (68)	3 (38)
**Ictal onset frequency**				
8 Hz or higher	88 (89)	14 (67)	24 (96)	6 (75)
**Ictal onset pattern**				
Fast spike trains	38 (38)	4 (19)	13 (52)	1 (13)
Low-voltage fast activity	33 (33)	10 (48)	7 (28)	5 (63)
Rhythmic slow activity	12 (12)	7 (33)	5 (20)	4 (50)
Electrodecremental activity	16 (16)	.	22 (88)	.
**Distribution of electrodes**				
Depth electrode only	47 (47)	2 (10)	15 (60)	2 (25)
Depth and medial strip electrodes	44 (44)	9 (43)	14 (56)	4 (50)
Lateral strip electrodes	3 (3)	8 (38)	3 (12)	3 (38)
Diffuse widespread	5 (5)	2 (10)	2 (8)	1 (13)
**Interhemispheric propagation**				
Early (within five seconds)	9 (9)	1 (5)	4 (16)	1 (13)
Late (above five seconds)	61 (62)	13 (62)	21 (84)	6 (75)
None	29 (29)	7 (33)	12 (48)	2 (25)

Values represent the number of events or cases (percent). † Shown are the number of cases exhibiting each electrographic feature in at least one recorded seizure event.

## Data Availability

The data presented in this study are available on request from the corresponding author. The data are not publicly available due to privacy and ethical restrictions.
